# Dealing with opposites as a mechanism of change in art therapy in personality disorders: A mixed methods study

**DOI:** 10.3389/fpsyg.2022.1025773

**Published:** 2022-12-30

**Authors:** Suzanne Haeyen, Johannes Ziskoven, Jackie Heijman, Evelien Joosten

**Affiliations:** ^1^Special Research Group Arts and Psychomotor Therapies in Personality Disorders, HAN University of Applied Sciences, Nijmegen, Netherlands; ^2^Department Scelta, Expert Center for Personality Disorders, GGNet Centre of Mental Health, Apeldoorn, Netherlands; ^3^KenVaK, Research Centre for the Arts Therapies, Heerlen, Netherlands; ^4^Department of Social Studies, University of Applied Sciences Utrecht, Amersfoort, Netherlands

**Keywords:** art therapy, personality disorder, opposites, mechanism of change, therapeutic factor

## Abstract

**Introduction:**

Personality disorders can be characterized by emotion regulation problems, difficulties in self-regulation and by dichotomous, black-and-white thinking. Dealing with opposites as a mechanism of change used by art therapists might be beneficial for people diagnosed with a personality disorder. This study examined the overall question if and *in what way* dealing with opposites in art therapy is a mechanism of change in achieving personal therapeutic goals.

**Method:**

A convergent parallel mixed-methods pilot study was performed among patients with a personality disorder (*N =* 32). Participants received four sessions of art therapy focused on opposites. They completed questionnaires on emotion regulation, positive and negative affect and sense of emotional balance before and after each session. Additionally, they completed a questionnaire on self-expression before and after the four sessions. Furthermore, 10 interviews (eight patients/two therapists) were conducted.

**Results:**

Quantitative results comparing baseline versus after the four sessions showed a significant change indicating that there might be a positive change regarding self-expression and emotion regulation (*t = −*2.45, *p* = 0.02, ES d = 0.30). A significant change was measured in acceptance of emotional responses (*Z* = −2.66, *p* = 0.01) and the state of emotion was rated as more balanced (*Z* = −2.19, *p* = 0.03). No further significant changes were found. Qualitative results showed that using opposites in art therapy often helped to gain insight, self-exploration and self-awareness and could facilitate confrontation as well as acceptance although sometimes it was (too) confronting.

**Discussion:**

Integration of conflicting emotions, behaviors, and thoughts were promoted by the explicit use of opposites and supporting coherent representation. Practice based recommendations are therefore to make more explicit use of dealing with opposites as a theme in art therapy. Also, we recommend more research on different mechanisms of change to refine the theory of change that provides an underpinning rationale and structure for art therapy. The results of this research should be regarded as exploratory given the small sample size and limited amount of therapy sessions.

## Introduction

Art therapy is a common treatment for people with a personality disorder (Haeyen, 2022). So far, research focused on studying the effectiveness of art therapy using predefined outcomes such as emotion regulation or mental health. *How* or *why* exactly it works has been insufficiently studied. Therefore, there is a need to better understand the process of change in art therapy. Change process research (CPR) inquires how or why treatment leads to change (e.g., Elliot, 2010). In (art) therapy it is important to gain insight into which factors are helpful and lead to therapeutic change (Bosgraaf et al., 2020; De Witte et al., 2021). This could lead to a better understanding and can ultimately increase the impact of the therapy.

Many art therapists offer art working methods based on the challenge of dealing with opposites: in material, instruction, themes or symbolism (e.g., soft versus hard, fluid versus solid, sensory/kinesthetic artwork versus cognitive symbolic). This is described in art therapy literature and art therapists consider working with these polarities as a therapeutic factor (e.g., Haeyen et al., 2015; Haeyen, 2018; Bosgraaf et al., 2020; De Witte et al., 2021) and a mechanism of change that would lead to an outcome such as improved emotion regulation. A mechanism of change is a theory-driven casual chain or sequence of events or processes (or mediating variables) that explain, in greater detail than factors or mediators alone, how or why therapeutic change occurs (Kazdin, 2009; Crits-Christoph et al., 2013; Avali and Sanderson, 2015; Pos and Choi, 2019; De Witte et al., 2021).

Dealing with opposites seems especially relevant as a mechanism of change among people diagnosed with personality disorders. Personality disorders are characterized by profound abnormal, unstable and dysfunctional cognitive, affective and interpersonal patterns that impair effective personal, social and professional functioning, paired with significant distress (American Psychiatric Association (APA), 2013). Instability of, and limited capacity for, emotion regulation and interpersonal contact are linked to a lack of integration (Young et al., 2003; Haeyen et al., 2015; Haeyen, 2018; Linehan, 2020). This results in the inability of the person to manage contradictions (Arntz and Ter Haaf, 2012). The coping style is characterized by dichotomous and ambivalent and extreme emotional responses; the typical “black-and-white” and “all-or-nothing” thinking. This coping response is often the result of previous trauma and evokes high perceived distress and over time develops into a persistent dominant personality trait (Veen and Arntz, 2000; Napolitano and McKay, 2007; Arntz and Ter Haaf, 2012; Oshio, 2012; Salters-Pedneault, 2021a,b).

Dealing experientially with opposites, conflicting emotions or contradicting needs might help to gain insight in the role these “opposites” play and how they can be worked with therapeutically in those with personality disorders (e.g., black-and-white thinking, splitting, impulsivity, lack of emotion regulation, diffusion, instability, and inner conflict; Arntz and Jacob, 2012; Arntz and Ter Haaf, 2012) Identifying and processing opposites and inner conflicts helps to manage these, so the person can move toward a more integrated, balanced and harmonious experience of self (Young, 2005; Arntz and Jacob, 2012; Claassen and Pol, 2014; Claassen and Pol, 2015).

Dealing with opposites to reach a more balanced way of dealing with emotions and impulses and aiming to improve identity integration, are features of Linehan’s Dialectical Behavioral Therapy and other therapeutic approaches (e.g., Riemann, 1961; Kris, 1993; Mokkenstorm and Van Tilburg, 1997; Tieleman, 2015; Van der Molen et al., 2015; Linehan, 2020). The inability to integrate conflicting needs, fears and urges leads to personality disturbances and impaired regulation and social functioning (Riemann, 1961; Kris, 1993; Mokkenstorm and Van Tilburg, 1997; Tieleman, 2015). Alternatively, conscious and active engagement with inner conflicts between opposites could allow for integration through creative synthesis in which paradoxical divisions and contradictions are resolved, informing a movement toward unity and healing (Johnson, 1991). In Schema Focused Therapy (SFT), developed for people diagnosed with personality disorders, this unity is associated with the Healthy Adult mode: the undivided identity in which all contradictory schemas and modes are integrated (Young, 2005; Claassen and Pol, 2014; Claassen and Pol, 2015).

In art therapy dealing with opposites, the ability to shape and process them, to reach a unified form, is a prominent theme (Malchiodi, 2011; Case and Dalley, 2014; Haeyen, 2018). Opposites, defined as the presence of completely different and opposing qualities, become visible and tangible in the therapeutic art making process (e.g., distance – proximity, autonomy – connection, big – small, hard – soft, open – closed, safe – unsafe, beautiful – ugly; e.g., Budde, 1989; Haeyen, 2007, 2011, 2018, 2019; Malchiodi, 2011; Case and Dalley, 2014; Hinz, 2015). Opposites in the artworks of patients could show patients’ possibilities for variation, differentiation and management of contrasts. Generally, movements of the patient in the creative process give an idea of their psychosocial possibilities, or, in other words, their rigidity or flexibility (Itten, 1980, 1989; Hinz, 2015; Haeyen, 2019). Contrasts and opposites are important image elements that can be used in the search for clarity, awareness and insight (Hinz, 2015). Opposites in form and process are analogous to life’s contradictions which play a prominent role in personality disorders.

Within art therapy, various approaches make use of opposites and are aimed at their integration. Art therapy combined with Dialectical Behavioral Therapy (DBT) for treatment of personality disorders focuses on the tension between opposites, applying processes of both acceptance and change (Haeyen, 2018; Linehan, 2020) moving through and with the opposites toward integration and harmony (Napolitano and McKay, 2007; Linehan, 2020; Salters-Pedneault, 2021a,b). Opposites as different sides of the self are also central in Gestalt Art Therapy and Jungian Art Therapy (Perls, 1973; Case and Dalley, 2014; Rhyne, 2016; Hamel and Hamel, 2020). The common theoretical model of the Expressive Therapies Continuum (ETC) works by exploring and balancing opposing components of information processing on different levels. On the Creative Level the integrated components bridge the inner experience and outer reality, allowing for personal growth, wholeness and health (Hinz, 2019).

Art therapy applies dealing with opposites aiming for integration, but it is indistinct how this works and what the effect is. How does artistic expression of inner conflicts and contradictions allow for exploration of these aspects? For instance, does the patient learn to cope with contradictions and create more coherence in feelings, behaviors and thoughts? In a previous study, personal integration was mentioned as a perceived effect of art therapy. This was defined as “to experience oneself more as a whole, more balanced and not so fragmented, torn, instable and dependent” (Haeyen, 2011, p. 40). Artistic expression also encourages healthy inner dialog by giving form to the various contradictory and opposing sides of the person (Malchiodi, 2011; Haeyen, 2019). Personal integration takes place when opposed feelings, thoughts and actions come together in one unified coherent representation (Rubin, 2001; Haeyen, 2019). Furthermore, the positive experience, playfulness, joy and creativity in art therapy combined with the shaping of expressive and felt artworks form the key aspects of psychological integration, growth and change (Chilton et al., 2015).

The question of *how* art therapy works with opposites can be answered with several descriptions. Art therapy has a connecting quality using creative processes in which different diffuse and unstable aspects of the patient with a personality disorder are brought together. Inner conflicts and contradictions between feelings and impulses are expressed in artwork in the form of opposites, making visible the fragmented and fragile self, promoting awareness, insight and self-reflection (Haeyen, 2011; Haeyen et al., 2015; Haeyen, 2018). Opposites offer an entrance to exploring the issues at hand in the creative process, and it is through opposites that the resulting shapes of that creative process can be perceived as a meaningful representation of the situation (e.g., Rhyne, 2016). Furthermore, art therapy is explicitly mentioned as affecting the processing of intrapsychic conflict (Jádi and Trixler, 1980) through the stimulation of integrating experiences (Haeyen, 2007; Van Vreeswijk et al., 2008; Gunther et al., 2009; Haeyen, 2011).

The prevalence of opposites as a main theme in personality disorders, psychological approaches and art therapeutic processes the question follows of whether dealing with opposites can be framed as a mechanism of change. In addition, how it functions and what its hypothetical effects are within art therapy in personality disorders. Despite this, hardly any research has been done on this topic and verification through research is needed (Haeyen, 2011; Federatie Vaktherapeutische Beroepen (FVB), 2017; Haeyen, 2019).

The aim of this study was to generate knowledge on dealing with opposites as a mechanism of change in art therapy for personality disorders, to refine the theory of art therapy (Hardy and Llewelyn, 2015). We examined the supposed mechanism of change of dealing with opposites in art therapy and what the effect in the session was of doing so. In addition, patients’ and art therapists’ perceptions of the added value of this supposed mechanism of change was assessed.

The quantitative part of this study was based on the hypothesis that by dealing with opposites in art therapy, patients will gain insight in their impaired integration or fragmentation and the role it plays in the problems they experience regarding emotion regulation, impulse control, social interaction and self-image. The qualitative research question was: How do patients and art therapists perceive working with opposites in art therapy and the meaning of this related to achieving their individual therapeutic goals?

## Method

This explorative research is a convergent parallel mixed-methods pilot study design including both quantitative and qualitative data measured at the same period of time, analyzed separately, and then triangulated (Creswell and Plano Clark, 2018; Dawadi et al., 2021).

### Participants

Participants in this study were adults (>18 years old) diagnosed with personality disorders clusters B and/or C (American Psychiatric Association (APA), 2013) receiving art therapy as part of four different out-patient multidisciplinary treatment programs at a specialized clinic for personality disorders. The weekly 1−1.5-h art therapy session was part of their standard treatment program. The standard program consists of multiple therapies: psychotherapy, societal rehabilitation, psychomotor therapy, music therapy and art therapy, next to daily tuning in and out sessions and joined lunches. Each therapy group consist of nine participants. The shared structure in the program consists of weekly themes based on emotion regulation, crisis skills, compassion skills, mindfulness and social skills. Based on the exploratory pilot study design, we aimed to include 20 to 25 patients who were willing to complete the pre-and post-session questionnaires. Furthermore, we included eight of the participating patients and the two art therapists to be interviewed. The sample of participating patients was chosen based on good representation of the target group, based on sex, age, personality disorder, and coming from the four different therapy programs.

### Procedures and ethics

Participants were recruited 2 weeks before the start of the first session and provided written informed consent. The art therapist explained the study and emphasized that participation was voluntary, and that withdrawal was possible at any moment. Comprehensible information and consent forms were handed out. After a week of consideration, patients could indicate whether they wished to participate or not. The study was executed in spring 2021 (February/May). Data were processed and analyzed confidentially. Audio recordings were destroyed after transcription. This study took place in the standard art therapy groups. The Ethical Research Committee of the HAN University of Applied Sciences has judged and accepted the study (ref.no. ECO 284.06/21). The study complies with the criteria of the Declaration of Helsinki on Ethical Principles for Medical Research Involving Human Subjects.

### Intervention

The art therapy assignments in the four weekly sessions were part of patients’ routine therapies. The four working methods with opposites as a theme were based on previously published intervention descriptions (see Haeyen, 2018; Haeyen, 2022). The working methods were discussed with the art therapists from the 3-day out-patient multidisciplinary treatment program for personality disorders who carried out the sessions and supervised by the research team. The intervention was offered in four successive weekly sessions with a duration of 1–1.5 h. The selection was based on the available practice-based description as well as consensus about effectiveness based on experiences and was also compiled in a way to include all levels of the ETC (Hinz, 2015). The art assignments were:

*Session 1*: *“Line drawing*.*” In this assignment the patients are asked to draw lines by following their momentary inner feelings and to subsequently exaggerate these lines*. *The patients are then asked to draw their attention to the opposite feelings and draw according to these*. *Finally*, *patients are asked to combine the two drawings to one meaningful picture*. *They can tear of glue the papers together to create one whole*.

*Session 2*:*“Mask inside out*.*” This session starts with a guided imagery to draw the attention to one’s inner world*. *After this*, *patients are instructed to paint a mask as a symbolic self-portrait of one’s inside* (*inner feelings*) *and outside* (*outside portrayal*), *depicting the inside out*, *and the outside in*. *Reflection afterward is focused on the message that it is okay to show your inner feelings*.

*Session 3*: *“Big and small self*.*” In this session*, *patients are asked to create clay figures of their big self and little self and to explore their relationships by creating different compositions of the clay figures placed in relation to each other*.

*Session 4*: *“Group paintings*.*” This assignment was a group painting themed ‘holding on – letting go’*. *Half the group works in a controlled way on one paper for 20 min*, *the other half freely expresses themselves on another paper*. *After 20 min*, *the groups switch to the other groups paper and continues in the style of the painting*.

### Demographic characteristics

Patient information was collected concerning gender, age, diagnosis, and scores of Global Assessment of Functioning (GAF), indicating severely impaired functioning (Haeyen, 2022) - high functioning (100) (American Psychiatric Association (APA), 2013).

### Quantitative instruments

Self-expression and Emotion Regulation in Art Therapy Scale (SERATS; Haeyen and Noorthoorn, 2021). This questionnaire is a one-factor instrument that measures self-expression and emotion regulation in art therapy. The instrument consists of nine items (e.g., “I am able to depict my feelings in art therapy,” “Making art is kind of an outlet for me.”), that are scored on a 5-point Likert (never true - almost always). Higher scores indicate greater self-expression and emotion regulation in art therapy. The reliability is excellent (Cronbach’s alpha of 0.94 and test–retest reliability r = 0.96). Correlations of the SERATS with the Emotional Expressivity Scale (EES), Emotion Regulation Strategies for Artistic Creative Activities Scale (ERS-ACA), and Healthy – Unhealthy Music Scale (HUMS) on the other hand has been reported as the highest for the ERS-ACA approach strategies with r = 0.69 in the patient sample and 0.81 in the student sample (Haeyen and Noorthoorn, 2021).

Positive and Negative Affect Schedule (PANAS; Watson et al., 1988; Peeters et al., 1996) measures affect using 20 items. The PANAS contains two subscales (positive, e.g., “Active” and negative, e.g., “Afraid”) consisting of 10 items each. Momentary presence of affect is rated on a 5-point Likert scale (very slightly or not at all – extremely). Higher scores subsequently correspond to higher positive affect (in the positive subscale) or higher negative affect (in the negative subscale). The PANAS has good internal consistency: Cronbach’s alpha = 0.89 for Positive Affect (PA) and 0.85 for Negative Affect (NA; Crawford and Henry, 2004), test–retest reliability: 0.79 for PA and 0.93 for NA (Ostir et al., 2005) and construct validity before (PANAS; Watson et al., 1988; Gratz and Roemer, 2004).

Difficulties in Emotion Regulation Scale (DERS; Gratz and Roemer, 2004; Dutch: Neumann et al., 2010) was used to measure multiple aspects of emotion dysregulation. To reduce test burden, only two of the six subscales were used (non-acceptance of emotional responses, e.g., “when I’m upset, I feel like I’m weak” and lack of emotional awareness, e.g., “I am attentive to my feelings”). Higher scores can be interpreted as experiencing more problems with this aspect of emotion regulation. Previously, a good reliability and construct validity were found (Gratz and Roemer, 2004; Fowler et al., 2014; Kökönyei et al., 2014; Ritschel et al., 2015). The Dutch version of the DERS was found to be reliable in an adult population with a Cronbach’s alpha of 0.80–0.90 for the different subscales (Slee et al., 2008).

Visual Analogue Scales (VAS; Wewers and Lowe, 1990). The VAS-scale is a non-specific measurement scale, consisting of a 100 mm long horizontal line (Wewers and Lowe, 1990). Responses are measured in centimeters. On the left side is the minimum score, on the right side is the maximum score. In this study, participants were asked to rate their state of emotion by two VAS-scales that indicated (1) from worst (0) to best state of emotion (100) and (2) totally disbalanced (0) to totally balanced (100) by placing a cross on the line.

**Figure 1:**
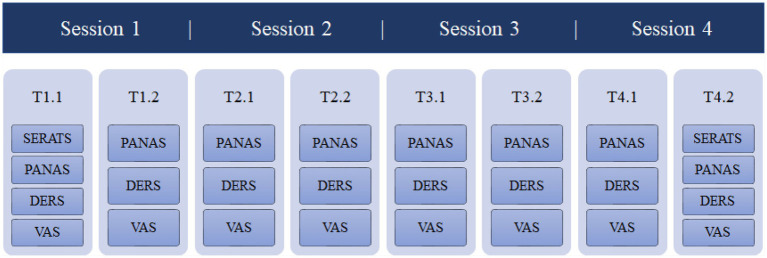
Questionnaires and measurements.

Patients completed the Self-expression and Emotion Regulation in Art Therapy Scale (SERATS) before session 1 and at the end of session 4, as it is indicated to be used after more than one art therapy session (Haeyen and Noorthoorn, 2021). Furthermore, questionnaires were completed before and after every individual session: Positive and Negative Affect Schedule (PANAS), two scales of the Difficulties in Emotion Regulation Scale (DERS) and two visual analogue scales (VAS; see Figure 1).

### Qualitative method

The VAS form also included an open question: “In which way did the session help you?.” Participants were asked this to gain insight into their motivation of their scores. Furthermore, this question gives information regarding the patients’ perception of the added value of working with opposites as a mechanism of change.

Interviews were conducted after session 4. These were semi-structured interviews. A topic list derived from the research questions (Taylor et al., 2015; Hennink et al., 2020) was used as a guide, and participants were encouraged to share their subjective stories using their own words to explain their personal experiences. Interviews lasted approximately 45 min and were carried out no longer than 4 weeks after completion of the intervention sessions. There was no selection for participation in the interviews, patients who offered to participate were included to a maximum of eight patients and two involved therapists. Interviews were recorded and transcribed. The artworks were present in the interview situation. Patients’ artworks were photographed after the interview. Interviews with the expert art therapists who carried out the intervention emphasized the patients’ perceived mechanisms of action in the offered work forms relating opposites.

### Data analysis

#### Quantitative data

Characteristics of the study population were summarized with descriptive statistics. Paired sample t-test was used to examine the mean difference on the SERATS before and after four sessions. In addition, Cohen’s d was calculated to determine the effect sizes (Cohen, 1988). Differences between before and after every session were tested using paired sample Wilcoxon signed-rank tests since these data were not normally distributed. We analyzed these three questionnaires before and after every session and not over all sessions. The SERATS is suitable for monitoring art therapy in the longer term, therefore, we asked patients to complete the SERATS before session 1 and at the end of session 4. Furthermore, questionnaires were completed before and after every session: PANAS, two scales of the DERS and two VAS questions (See Figure 1). All statistical tests were two-sided, with a *p*-value of 0.05 or less considered to indicate statistical significance. All analyses were performed using SPSS (Corp, 2020).

#### Qualitative data

The written elucidation of the VAS score and the interview transcripts were analyzed through qualitative analysis and Grounded Theory (Charmaz, 2006; Denzin and Lincoln, 2011). The data was coded inductively using a round of open coding and a second round of axial coding, resulting initially in a code tree with 44 codes. Finally, the following 9 core themes were formulated: inside versus outside, freedom versus structure, inner conflicts, gaining self-insight, self-expression, emotion regulation, dialog between opposing sides of self, sense of balance, and self-acceptance.

The codes were then resorted through a third round of selective coding. This analysis allows for the start of development of a theoretical framework regarding the research question about opposites as a mechanism of action in art therapy for personality disorders. The photos of artworks were used to enrich the qualitative narrative and serve as descriptive and illustrative material in the process of qualitative analysis.

#### Integration of quantitative and qualitative data

Most common noted themes of patients’ VAS scores were coded and counted. These themes were then compared to the quantitative measurement of the VAS, to see if the written elucidation matched the quantitative data from the VAS itself. Also, quantitative results were combined and compared with the qualitative results.

## Results

### Demographics

Thirty-nine patients were eligible of whom seven were not willing to complete the questionnaires. A total of 32 patients were included. Gender, age, diagnosis, and scores of GAF (American Psychiatric Association (APA), 2013) are shown in Table 1. The largest group of the participants (37.5%) had a GAF score of 50, indicating severe symptoms, such as suicidal thoughts or severe, obsessive rituals, and being unable to keep a job.

**Table 1:** Demographics of the participants (*N* = 32).

Gender	Female (25)	78.1%
	Male (7)	21.9%
Age (yrs)	Mean 37.53 (SD = 9)	Range 24–60
Diagnosis	Unspecified PD (301.89/9)	37.5%
	Borderline PD (301.83)	31.3%
	Avoidant PD (301.82)	28.1%
	Obsessive–compulsive PD (301.4)	3.1%
GAF-score	40	12.5%
	45	25%
	50	37.5%
	55	15.6%
	60	9.4%

The eight patients who were interviewed were representative of the wider sample with on average 35 years of age (range 25–45); 6 females (75%) and 2 males; and diagnosed with Unspecified PD [5x], Borderline [1x], and Avoidant/dependent PD [2x].

Two therapists who were interviewed were one senior and one junior art therapist (both female, 55 and 25 years, working experience 30 and 1.5 years).

### Overall quantitative results

A statistical difference was measured regarding self-expression and emotion regulation in art therapy measured with the SERATS (*t* = −2.45; Arntz and Jacob, 2012, *p* = 0.02). SERATS scores increased significantly between start and end of four art therapy sessions indicating a positive effect of making art in a healthy way (see Table 2). Significant changes were found within session 2 and 3 based on the results on the DERS and the VAS-scale (see description per session and Table 3).

**Table 2:** Self-expression and emotional regulation in art therapy (SERATS) before and after the four sessions (*N* = 21).

	Before (T1) Mean (SD)	After (T8) Mean (SD)	*t*-value, *P*-value	ES d
SERATS	26.81 (4.55)	28.19 (4.68)	−2.45, 0.02*	0.30

* *p* < 0.05.

**Table 3:** Participants’ indicated state of affect (PANAS), emotional dysregulation (DERS), and state of emotion (VAS) - before and after session.

Session number		N^a^	Before session Mean (SD)	n	After session Mean (SD)	Z-value, *p*-value
Session 1	PANAS					
	Positive	26	20.35 (5.98)	26	21.08 (8.61)	−0.86, 0.39
	Negative	27	25.19 (9.17)	26	24.00 (9.56)	−0.50, 0.55
	DERS					
	LEA^b^	27	13.96 (2.16)	25	14.04 (2.13)	−0.31, 0.76
	NER^c^	27	20.07 (5.73)	25	19.44 (6.99)	−0.98, 0.33
	VAS					
	Balance	27	3.67 (1.90)	26	3.77 (2.20)	−0.24, 0.81
	Current mood	27	3.74 (1.53)	26	3.96 (2.13)	−0.33, 0.74
Session 2	PANAS					
	Positive	27	19.22 (5.50)	27	20.22 (6.36)	−1.52, 0.13
	Negative	27	26.74 (9.80)	27	26.67 (9.03)	−0.78, 0.44
	DERS					
	LEA	27	14.07 (1.92)	27	13.74 (1.70)	−0.37, 0.71
	NER	27	21.52 (5.39)	27	20.07 (6.15)	−2.66, 0.01*
	VAS					
	Balance	27	3.37 (2.01)	27	3.48 (1.70)	−0.66, 0.51
	Current mood	27	3.56 (1.85)	27	3.37 (1.89)	−0.27, 0.79
Session 3	PANAS					
	Positive	25	20.76 (8.27)	25	20.04 (6.19)	−0.77, 0.44
	Negative	25	25.04 (8.29)	25	24.04 (9.76)	−1.11, 0.27
	DERS					
	LEA	23	13.61 (1.64)	24	13.17 (1.49)	−1.30, 0.19
	NER	23	19.43 (7.21)	24	19.54 (6.81)	−0.44, 0.66
	VAS					
	Balance	25	3.00 (2.42)	25	3.84 (2.48)	−2.19, 0.03*
	Current mood	25	3.52 (2.22)	25	3.64 (2.55)	−0.20, 0.84
Session 4	PANAS					
	Positive	23	22.00 (6.66)	21	22.67 (6.48)	−0.44, 0.66
	Negative	23	23.70 (9.03)	22	21.86 (8.87)	−1.11, 0.27
	DERS					
	LEA	22	13.36 (2.44)	20	12.95 (1.79)	−0.92, 0.36
	NER	22	19.36 (7.02)	20	18.90 (7.00)	−1.45, 0.15
	VAS					
	Balance	23	3.70 (2.38)	22	3.86 (2.46)	−0.17, 0.86
	Current mood	23	3.96 (2.12)	22	4.50 (2.52)	−1.30, 0.20

* *p* <0.05. ^a^The different numbers of participants are explained by the absence of different participants each session. The presence of participants in each of the four sessions varied between 84.4 and 62.5%.

^b^LEA, lack of emotional awareness.

^c^NER, non-acceptance of emotional responses.

### Overall qualitative core themes

Perceived effects of dealing with opposites as described by patients and therapists in the interviews and the open question were summarized in core themes: inside versus outside, freedom versus structure, inner conflicts, gaining self-insight, self-expression, emotion regulation, dialog between opposing sides of self, sense of balance, and self-acceptance. In the following text these are elaborated. Furthermore, the perceived added value of dealing with opposites in art therapy was also formulated. To zoom in, the qualitative responses of the patients per session were collected. Finally a synthesis of quantitative and qualitative results per session is presented as well as an overarching synthesis of pre and post quantitative and qualitative results.

Patients mentioned the opposite of ***inside* versus *outside*** as a theme and stated that the contrast between what is felt inwardly and expressed outwardly is often strong. Mostly, opposites were prevalent when perceived negative emotional sides were hidden in favor of thoughts. They expressed a deep personal tension between who they feel they are deep down, and how they think they need to present themselves (in thought, feelings and/or behavior). Often, vulnerability and pain were hidden and a controlled persona was shown, consisting of mainly coping strategies.

**Figure 2:**
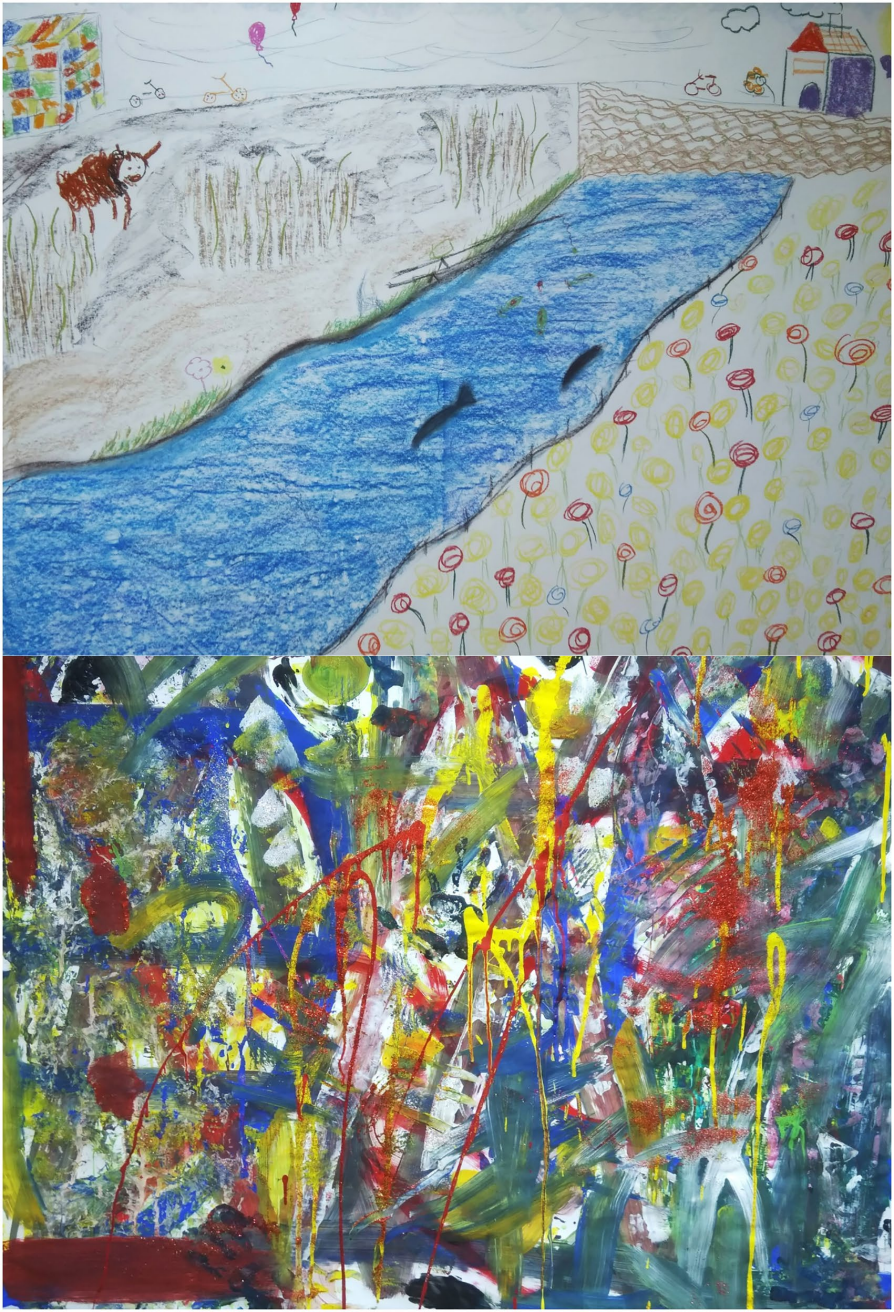
Group paintings.

Patients reported that they projected their experienced dividedness and struggle onto the contrasting artistic expressions in their artwork. Consequently this opened a space in which an inner dialog between the inner experience and outward form raised. Patients mentioned the importance of color contrast when seeking to accurately expressing feelings, for example light and dark color, and colorfulness versus black and white (see Figure 2).

“In my opinion color contrasts say a lot about the feelings that I show.”

- Patient 1, male, 37 yrs. (assignment session 1: Line drawing).

The opposition of ***freedom* versus *structure*** came forward. Patients mentioned both the joy of liberation when art making without expectation or plan, and the security found in structure and clarity. Therapists mentioned that there is a need for balance between the opposing and complementary qualities of letting go and holding on. According to the therapists, exercises in art therapy that play with these opposites, allowing the patient to move between the extremes, can stimulate alternative experiences that increase flexibility. Patients explained that fear of doing the wrong thing or losing control blocks this movement. Therapists, in their turn, through offering working methods based on dealing with opposites aimed to encourage patients to confront their fears and explore the divisions and contradictions in their thoughts, feelings and behavior. Through this creative engagement, patients learn to bear these opposites and reflect on what is going on so they can deal with it better.

Patients explained the origin of their ***inner conflicts***, talking about experiences of neglect, criticism, or abuse, not knowing how to deal with their vulnerable, insecure, and desperate sides, suppressing their feelings to “please” others to receive the love, acceptance, encouragement and support they crave and need for healthy development. Patients stated that they have no clear sense of who they really are, always suppressing and adapting, feeling enormous pressure to meet expectations and experiencing a fear of rejection.

**Figure 3:**
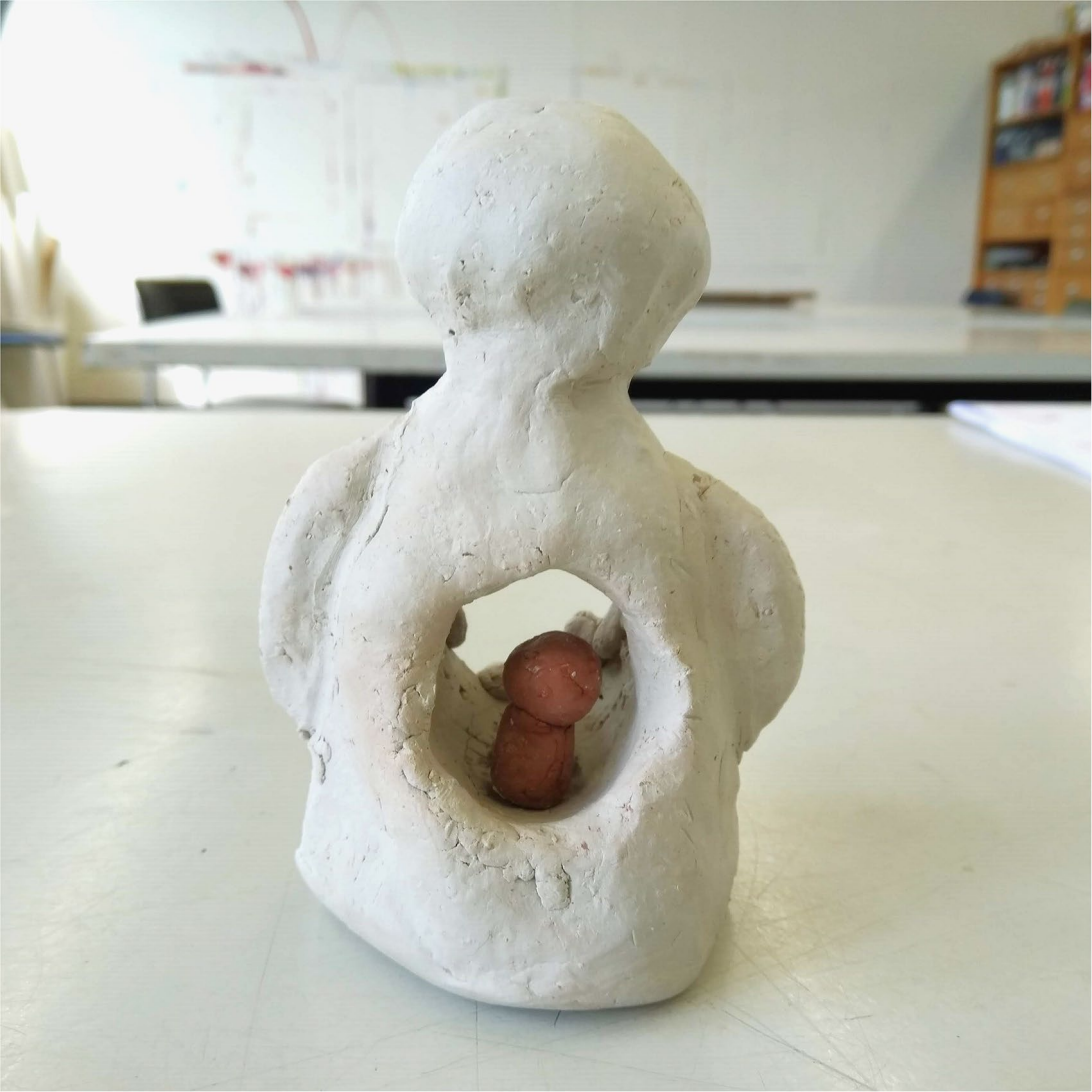
Big self and little self.

The art therapists stated that opposites can be found everywhere in artwork and process because opposites are inherent to the way we perceive shapes, colors, movements, themes, and the associated narratives and experiences. Therefore, they stated that working with opposites offers an entrance for ***gaining self-insight*,** which is also stated in the following quote and accompanying Figure 3.

“Working with opposites invites to get to know both sides, and thus develop a bit of flexibility to better deal with them.”

- Art therapist 2 (assignment session 2: group paintings).

**Figure 4:**
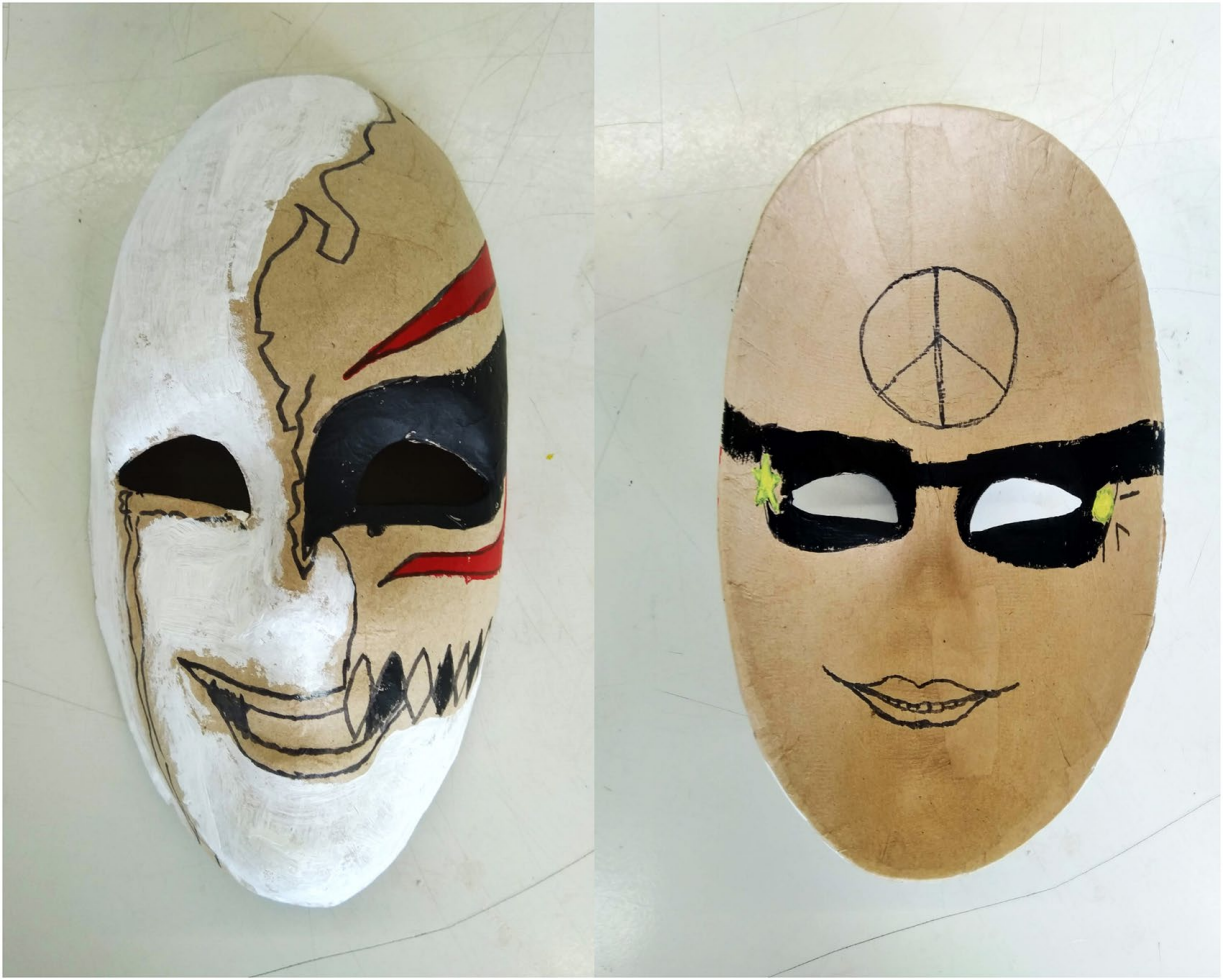
Mask inside out.

The expressive work is the point of departure for questioning oneself and the way one relates to opposites. Patients mentioned the clarity of a contrasting image that makes self-observation more concrete, tangible and thus understandable. They stated that these contrasts aid in the process of expression, communication, and clarification that leads to insight into what is going on. Patients mentioned that dealing with opposites challenged them to tune into vulnerability and pain and make visible and intelligible the parts of the self which are hidden (see Figure 4).

*“Working and dealing with opposites helps make visible what is hidden – seeing the inside of how you feel*. *By seeing it*, *it becomes more real*, *and then you can better understand it*.*”*

- Patient 6, female, 35 yrs. (assignment session 3: mask inside out).

Patients stated that creative work with opposites, despite the fact that this could be confronting, fear-provoking and stressful, paved the way of exposing their hidden sides and come to peace with these. Patients mentioned how art therapy allows for ***self-expression*** and ***emotion regulation*** through the balance between expression and structure, preventing potentially overwhelming exposure. Rather than experiencing discomfort, some patients expressed feeling comforted by the sense of acceptance that came with making visible what they normally suppress.

*“The moment you let it be there – you start to feel it – you can learn to deal with it*.*”*

- Patient 6, female, 35 yrs.

Patients mentioned that bringing into view and integrating all parts of the self is difficult. Images that contain both polarities of the opposites expose the “ugly” and undesirable aspects of the personality and the conflicts patients often experience, which was often perceived as frustrating and disheartening. Therapists mentioned that integrating opposites takes time, and that the initial discomfort is necessary to open up the ***dialog between the opposing sides of the self*.** They elaborated on how the dialog takes place in the shapes, lines, faces, movements and representations expressed in the artwork. Patients described how they seek a personal balance, finding reference points in the creative experience as they reflected, analyzed, and ascribed meaning to their artworks.

*“Creative exercises help me to show myself from within – I give myself an answer to question of what I am actually feeling*.*”*

- Patient 3, female, 29 yrs.

When asked what they felt working with opposites accomplishes, patients mentioned a ***sense of balance*** and ***self-acceptance*.** They expressed a desire to feel less divided, less conflicted, and less contradictory. They mentioned self-expression and integrated representation in artwork as important factors that contributed to or elaborated on this desire. Accepting opposites within the self, learning to deal with them and wanting to change them seemed equally prevalent. However, all patients hoped to decrease their experienced opposites, so they might feel more regulated, integrated, and in control.

#### The perceived added value of dealing with opposites in art therapy

*“It is helping for me*. *It clarifies*. *I become aware of the things that I hide*.*”*

- Patient 4, female, 45 yrs.

Therapists mentioned that besides the general value of experiential art therapy, having to deal with opposites specifically stimulates awareness and insight, promoting self-exploration and understanding. Some patients appreciated the experiential process and mentioned how engaging in art making supported their process and how working with contrasts helped them gain insight and acceptance. To others it had limited additional value over what they already knew.

*“I’m not saying that I directly have all the answers and solutions and that I have succeeded*, *but it does give me insights*.*”*

- Patient 8, male, 39 yrs.

**Figure 5:**
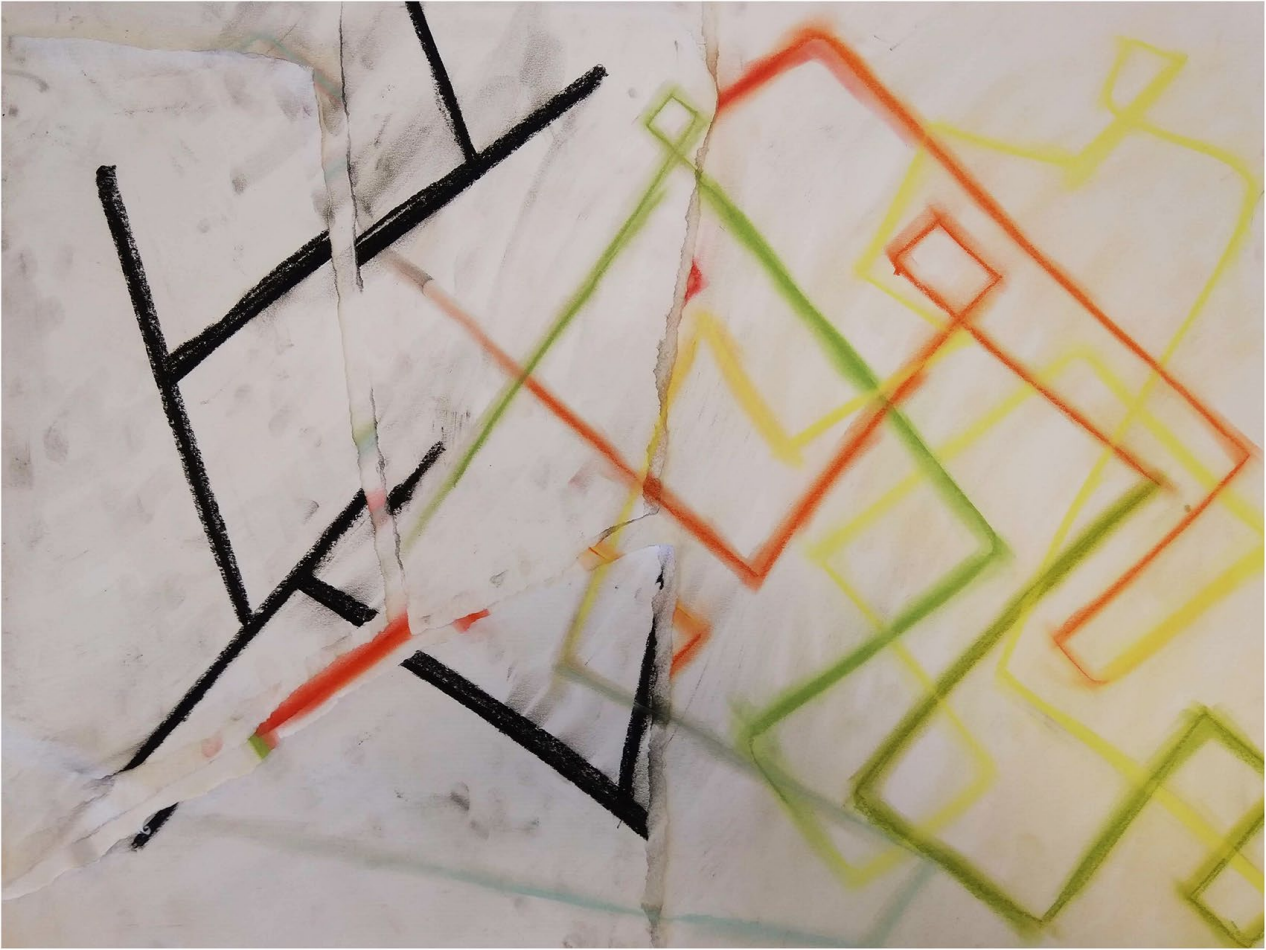
Abstract line drawing.

On the same theme, the therapists suggested that experiential and creative expression adds to the deepening of patients’ insight and self-awareness by offering both reflective distance and felt experience in dealing with opposites. They added to this that reaching a sense of wholeness, coherence and acceptance through creatively bringing together opposites is easier for some than for others (see Figure 5). Often this can be considered a therapy endpoint.

*“Putting it all together in one form does make me think: okay but*... *maybe I actually AM whole*.*”*

- Patient 2, female, 42 yrs. (assignment session 4: big and small self).

Some patients mentioned that dealing with opposites is only a pleasant and valuable experience if they were capable to integrate the opposites into a whole. When inner conflict and contradictions were not integrated, working with opposites was perceived as confronting and unpleasant, emphasizing the personal fragmentation and instability the patients’ experience.

Therapists interpreted this as different parts of the therapeutic process, some of which are more confronting, whilst others are more soothing and affirming. They stated that on one hand it is about bearing the painful issue, while on the other hand it is about healing; “Successful therapy needs both.” Therapists stated that ultimately, only when patients can transcend the apparent conflict between opposites can they begin to heal and realize their wholeness and authenticity. Even though dealing with opposites may promote insight, understanding, acceptance and personal integration in patients who are `on track’ in their treatment process, for patients it is quite common to find the process also distressing initially. Patients mentioned painful experiences of instability and dividedness and discouraging self-criticism and doom-thinking when unable to integrate opposites in an artwork which might lead to heightened emotionality and difficulties in self-regulation. Therapists indicated that it is their task to mitigate and manage the balance between confrontation and comfort in this process:

*“Especially when making visible the most hidden and least accepted parts that are harshly criticized and rejected by the patients*, *this is crucial*. *In the end*, *confrontation with opposites can be a powerful tool in working toward a more healthy*, *integrated and whole sense of self for patients with personality disorders*.*”*

- Therapist 1.

#### Results per session

**Figure 6:**
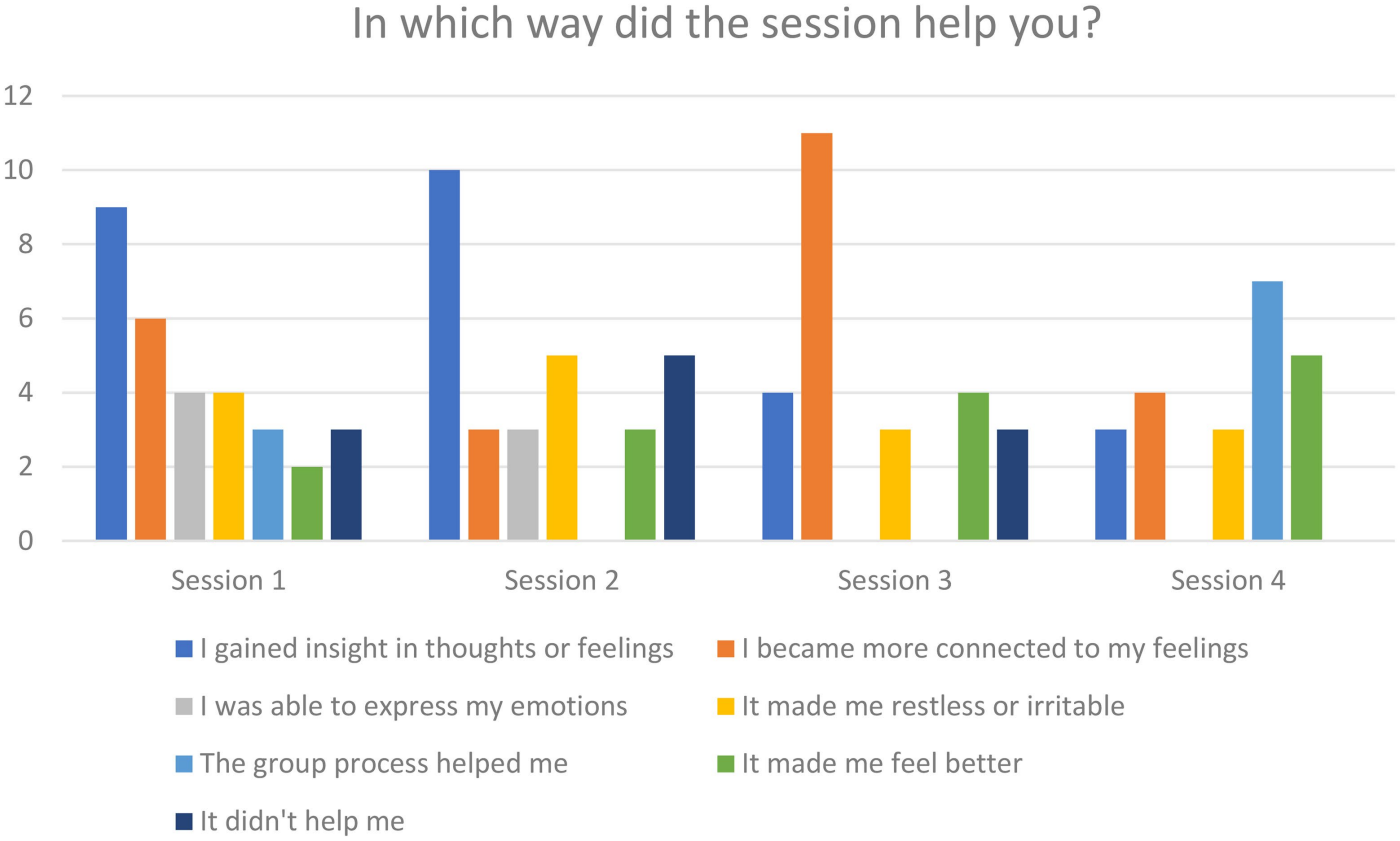
Responses to the open question per session (1–4) grouped by theme (*N* = 27/27/25/23).

Patients’ qualitative responses per session were extracted from the open question on the VAS-form as well as from the interviews. Patients’ responses to the open question (e.g., “in which way the session helped”) were grouped into themes as shown by Figure 6.

**Session one** (line drawing) was described as gaining insight into feelings and thoughts. In the interviews, patients mentioned gaining insight from the first session, but were less explicit in the specific content and effects of the session. From the responses to the open question, it seemed to be a confronting exercise to deal with their first action and experiencing the opposite action as well. Some participants became aware from the result of this exercise that they were not doing so well. This led to some responses that it was not “helpful” although also multiple participants mentioned coming closer to feelings and expressing them. The quantitative data of session 1 showed no significant results (see Table 3).

*“I actually feel worse than before the session because now it became visible how I could be feeling: the opposite of how I feel right now*.*”*

- Patient 10, female, 42 yrs.

**In session two** (mask making), the most frequently mentioned helpful aspect was gaining insight into thoughts and feelings. Having to deal with the contrast between the inside and the outside of the mask seemed to be the trigger for this, but the contrast was also confronting and could lead to irritation. Those respondents felt that this session did not help them. For others, being in the moment and making positive discoveries in their artwork and helped them feel better. In the interviews, the second session was mentioned as increasing awareness and acceptance of the ‘hidden’ and rejected parts of the self after seeing their contrasting inside and outside expressed in the mask. The quantitative data of session 2 showed a significant difference concerning emotional dysregulation measured by DERS. Participants indicated less non-acceptance of emotional responses after session 2 (Z = 2.66, *p* = 0.01). No other significant differences were found (Table 3).

*“I gained insight not only through my own mask but also the masks other patients often wear*. *This helped me to gain insight and understanding*.*”*

- Patient 12, male, 37 yrs.

**In session three** (clay figures) most participants mentioned feeling more connected to their feelings. Participants mentioned calmness in their bodies.

*“I feel more peace in my body*. *The strong side of me has ‘put a hand’ on the vulnerable side of me*.*”*

- Patient 32, female, 35 yrs.

Some participants mentioned the visibility of their strong and vulnerable side in their artwork. Based on the interviews, the third session emerged as mostly leading to a realization of needs to feel safe, cared for, held and at peace. Being confronted with figures of the big and little self and how the fear, sadness, pain and loneliness of the vulnerable side could be soothed by the closeness of a caring figure, aroused feelings of consolation and hope. Some respondents do not report this session as “helpful.” The most explicitly mentioned aspect was the importance of the sense of “allowing the vulnerable side to just be there," which was aided by the supporting stronger side. In the quantitative outcome measures, participants rated their state of emotion as more balanced after session 3 by the VAS-scale (Z = −2.19, *p* = 0.03). No other significant differences were found (Table 3).

**In session four** (group exercise) the group process was indicated as most helpful because participants felt more joyful afterward. Some of them mentioned that being part of a group helped them to stop thinking and be in the moment, and the session was generally experienced more lightly. “Working together made me feel better” was noted a few times. Based on the interviews, patients mentioned initially feeling somewhat inhibited by perfectionism, fear of failure, and the pressure to perform well. However, the group process; seeing others have fun expressing themselves and inviting self-expression in other group members, as well as the expressive appeal of the assignment lowered these inhibitions, allowing for a more flexible way of dealing with the opposites with more free expression and fun. The quantitative data of session 1 showed no significant results (see Table 3).

#### Synthesis of overarching pre and post results

A significant positive change was found regarding more expression and insight after the four sessions compared to the first point of measurement, before session 1. This is not a causal effect because other interventions besides art therapy took place during this four-week period. In the interviews the perceived overarching changes based on dealing with opposites were generally expressed in terms of stimulating emotional awareness and insight, promoting self-exploration and understanding, allowing expression and making visible the rejected and repressed emotional sides of the self, and promoting a sense of emotional balance and acceptance. Some patients reported that they had not reached this sense of balance yet, but that the expressed opposites emphasized the visible and tangible insight into their suffering. Dealing with opposing contrasts helped to create clarity promoting self-insight. However, this could lead to patients feeling either positive or negative emotions depending on the phase of their individual process.

#### Synthesis of quantitative and qualitative results

##### Session 1

Quantitative data did not show notable results, whilst the written responses to the open question indicated that the session was helpful for gaining insight. Patients also mentioned gaining insight in the interviews but were less specific about what was helpful about the insights.

##### Session 2

The second session resulted in significant decrease in non-acceptance of emotional responses, which seemed to correspond to gaining insight into thoughts and feelings. This is a causal effect based on the pre−/post-session measurement. The written responses to the open question emphasized gaining cognitive insight, whilst the interview responses elaborated on how the confrontation with both sides of the mask helped patients gain acceptance of their repressed sides. However, this could lead to patients feeling either positive or negative emotions depending on the phase of their individual process.

##### Session 3

The quantitative results showed that participants felt significantly more balanced after working with the clay. This is a causal effect based on the pre−/post-session measurement. Also, the written responses to the open question showed increased emotional connection to themselves. In the interviews patients mentioned how they felt connected with both their vulnerable and strong sides, which increased a sense of safety and holding, and contributed to a sense of inner balance.

##### Session 4

There were no noticeable quantitative results. However, the written responses to the open question indicated patients feeling better and being helped by the group process. In the interviews patients mentioned the support and joy of collaborating in the group. Mutual invitation and stimulation to express promoted a freer self-expression and increased fun.

## Discussion

In this study, we examined the supposed mechanism of change of dealing with opposites in art therapy and what the effect in the session was of doing so. In addition, patients’ and art therapists’ perceptions of the added value of this supposed mechanism of change was explored. Doing so, we aimed to generate knowledge on the application and effect of the mechanism of change of dealing with opposites in art therapy for personality disorders.

The results from this study partly support these hypotheses. These indicate that dealing with opposites might have a positive effect on self-expression and emotion regulation, with more acceptance of emotional responses and a more balanced state of emotion. Whether a positive change was taking place or not is nuanced. The quantitative results in this study show the average of the reported experiences, the qualitative results show a rich description. Both results complement each other. Respondents recognized opposites as an important theme in their lives, as seen in contrasts like `inside versus outside’, `freedom versus structure’, which have parallels with their felt inner conflicts. Inside and outside symbolizes the conflict between inner and outer world, dealing with emotions and expectations of oneself or as dealing with oneself as perceived of others. Freedom versus structure in artwork is the search for balance in the conflict between art making without expectation or plan (liberation), and with structure and clarity (security). These conflicts seem to be analogous to inner conflicts, having an origin in patients’ past experiences of neglect, criticism or abuse or to their challenge to deal with the outside world.

Perceived effects of dealing with opposites as mentioned by patients and therapists were: gaining self-insight (the clarity of a contrasting image makes self-observation more real, tangible and thus understandable), allowing for self-expression and emotion regulation (through the balance between expression and structure), creating a dialog between the opposing sides of the self (facilitated by the patients’ confrontation with own felt opposites), and creating a sense of balance and self-acceptance. A perceived added value or helping factor of explicitly having to deal with opposites was stimulation of awareness and insight by offering both felt experience and reflective distance. Some respondents also reported that confrontation was not always perceived as helpful and leading to positive experiences. For some, the confrontation meant a desired therapeutic yield, for others this meant an unpleasant feeling that was rather not felt. It was noted that ultimately, only when patients can transcend the apparent conflict between opposites can they begin to recover and realize their wholeness and authenticity. Integrating opposites into one coherent representation comes forward as assisting this process toward personal integration.

Opposites have been an integral part of psychological theories for decades (psychoanalysis, Gestalt therapy, DBT). Also, in recent theories in the neuropsychiatric balance between being too sad or too happy (manic) is being pursued by homeostasis (Simonetti et al., 2020) and theories about emotion regulation are used in trauma psychotherapy for hyper or hypo arousal (Jenkins, 2018). Also, in other art-based therapies working with opposites is common. For instance, in drama therapy role theory is applicated with protagonists and counter roles (the antagonists) and with high/low status roles (Landy, 2009). In music therapy, internal conflicts can be expressed by strong use of musical dynamics by playing something extremely loudly or quietly (Strehlow and Lindner, 2016). In dance therapy, complementary qualities of movements are used to restore balance (Angeletti, 2016). In psychomotor therapy, regulation is practiced between externalizing and internalizing (Boerhout and Van der Weele, 2007). These applications are in line with the findings of this study and although opposites have been an important part of psychological theories for a long time, this is the first clinical study in art therapy that focuses on the specific theme of dealing with opposites as a mechanism of change. Another strength of this study is the mixed method design. Data was retrieved from both quantitative questionnaires and qualitative interviews of patients and therapists and written open answers. This resulted in the possibility to compare different types of data with a broad scope of information on the context and experiences.

A study limitation is the small sample size and the involvement of only two therapists, as this was realistically feasible. Although saturation might occur in under 12 interviews (Guest et al., 2006), we acknowledge the limits of this small sample size. Therefore, these results should be regarded as exploratory. A second limitation is the limited number of sessions. Expectations of effects from only four sessions should be modest given that personality disorders have been longstanding before therapy. In addition, we cannot claim the long-term effectiveness of the sessions because they were not studied. On the contrary, based on the present results, a target group of relatively milder or healthier patients could possibly profit even more. However, in the target group of patients diagnosed with personality disorder the dichotomy and dysregulations are the main characterizing themes and therefore explicitly dealing with opposites in art therapy might be especially beneficial.

The different mechanisms of change in art therapy and their potential benefits are receiving growing attention. Specific mechanisms of art therapy have been studied within different populations (Czamanski-Cohen and Weihs, 2016; Armstrong and Ross, 2020; Bosgraaf et al., 2020; Schnitzer et al., 2022). These studies have contributed to the knowledge base of art therapy and can offer specific leads for future research. However, this area of research is also complex. For instance, common and specific factors are significantly correlated and are thus difficult to differentiate (De Felice et al., 2019). A strong alliance between the therapist and patient is an important common factor (Wampold, 2010) which may be very helpful for a specific factor to be able to be effective.

Results of this study identified dealing with opposites as a specific mechanism of change in art therapy leading to relevant outcomes. Practice based recommendations are therefore to make explicit use of dealing with opposites as a theme in art therapy. Many art therapists may already use interventions based on opposites, but improved insight into the effect and value of working with opposites may help to develop more targeted interventions and to improve effectiveness of their therapy.

We also recommend more research on the therapeutic factors and mechanisms of change to refine the theory of change that provides an underpinning rationale and structure for art therapy. This study provides a start to building a theory of change that is supported by evidence. In these future studies we suggest making combinations with existing psychological theories, for example regarding emotion regulation. We also recommend using questionnaires aimed at the social aspects when investigating group interventions of art therapy to be able to identify the mechanism of change involved. More specifically, interventions that can be investigated further include the following; the use of clay combined with symbolic figures that represent contrasting sides of the self, seemed to be relatively effective in restoring personal balance; the social aspect in therapy seemed important for mood improvement; and the combination of different, contrasting ETC levels (e.g., sensory, affective or symbolic layer) might increase personal integration. As stated by Hinz (2015), Integration of layers creates the creative component. These aspects could be studied more closely.

Dealing with opposites offers a playing field for dialog, confrontation, comfort, experimentation with alternatives and regulation. Especially for people with personality disorders, dealing with opposites could be useful in the different phases of their therapeutic process. Inner conflict and contradiction are prevalent in people with personality disorders and seems to be related to basic needs not being met. This often results in a disturbed personal development presenting with instability, problems with emotion-regulation, and lack of coherence and the absence of a sense of wholeness. In dealing with opposites, we might create acceptance of opposites or find a new balance in burden and capacity.

By identifying dealing with opposites as a mechanism of change toward improved self-expression and emotion regulation, we have contributed to refining the theory of change underpinning a rationale and structure for art therapy. This could inform development of more effective training and supervision on dealing with opposites as an effective therapeutic mechanism of change that is supported by evidence.

## Data availability statement

The raw data supporting the conclusions of this article will be made available by the authors, without undue reservation.

## Ethics statement

The studies involving human participants were reviewed and approved by Ethical Research Committee of the HAN University of Applied Sciences (ref.no ECO 268.05/21). The patients/participants provided their written informed consent to participate in this study. Written informed consent was obtained from the individual(s) for the publication of any potentially identifiable images or data included in this article.

## Author contributions

SH initiated the research on opposites, responsible for reviewing the overall article and rewriting main parts of the introduction, method, results, and discussion. JZ is responsible for the qualitative data collection of interviews, co-writing main parts of the introduction, method, and results, and analysis of the interviews. JH is responsible for co-writing main parts of the introduction, method, results, and discussion, analyzing the VAS open question, reporting these results, and checking the references of the article. EJ is responsible for the quantitative data analysis, reporting the quantitative data analysis method and results, and contributed in reviewing the overall article. All authors contributed to the article and approved the submitted version.

## Conflict of interest

The authors declare that the research was conducted in the absence of any commercial or financial relationships that could be construed as a potential conflict of interest.

## Publisher’s note

All claims expressed in this article are solely those of the authors and do not necessarily represent those of their affiliated organizations, or those of the publisher, the editors and the reviewers. Any product that may be evaluated in this article, or claim that may be made by its manufacturer, is not guaranteed or endorsed by the publisher.
